# Effect of Neoadjuvant Chemotherapy on the Serum Levels of Bone Turnover Markers in Women with Early-Stage Breast Cancer

**DOI:** 10.1371/journal.pone.0126053

**Published:** 2015-04-29

**Authors:** YangYang Chen, GuoBin Xu, Feng Yang

**Affiliations:** 1 Department of Clinical Laboratory, Peking University Cancer Hospital and Institute, Beijing, China; 2 Department of Breast Surgery, Peking University Cancer Hospital and Institute, Beijing, China; The First Affiliated Hospital with Nanjing Medical University, CHINA

## Abstract

**Background:**

To evaluate effects of neoadjuvant chemotherapy on the bone turnover markers of preoperational breast cancer patients.

**Methods:**

Forty-one breast cancer patients (29 premenopausal and 12 postmenopausal) and 60 healthy women (30 premenopausal and 30 postmenopausal) aged 30-64 years, were evaluated for their bone status. Serum levels of the bone formation markers PINP and BAP, as well as the resorption markers ICTP and β-Crosslaps in addition to E2, FSH, 25(OH)D and PTH were measured at the initial diagnosis and at 24 hours after each four chemotherapy cycles. BMD T-scores were determined in 12 patients 6 months after the neoadjuvant chemotherapies.

**Results:**

The baseline levels of both bone formation and resorption markers in premenopausal patients were higher than in premenopausal healthy women (p<0.05), while no statistic difference was observed between postmenopausal patients and postmenopausal healthy women. Regardless of the menopausal status, chemotherapy increased the ICTP and β-Crosslaps levels (p<0.05), but decreased the BAP and PINP levels (p<0.05), the later one significantly more with Taxane medication (p<0.01, p<0.05). Chemotherapy caused significant decreases of 25(OH)D levels in premenopausal (p<0.01) and postmenopausal (p<0.05) patients, however, did not affect the PTH concentrations. In premenopausal patients the E2 level decreased, while the FSH level increased after chemotherapy (p<0.05). Patients with pronounced ICTP and β-Crosslaps combined with reduced BAP and PINP serum concentrations after neoadjuvant chemotherapies were prone to develop osteoporosis 6 month later.

**Conclusions:**

Neoadjuvant chemotherapy appeared to promote bone resorption and inhibit bone formation in both postmenopausal and premenopausal early-stage breast patients.

## Introduction

Breast cancer is the most common malignant tumor in women. With the advancement of treatment, the breast cancer survival rate has dramatically increased and patients’ life extended. As a result, maintenance of the bone health became one of the most important factors for high quality life of patients. Cancer therapies employing chemotherapeutic reagents such as aromatase inhibitors [1] were shown to adversely affect bone health, increasing the risk of osteoporosis and even fracture. Ovarian failure caused by chemotherapy can accelerate bone loss in breast cancer patients[2–5]. Some chemotherapeutic drugs including cyclophosphamide, methotrexate, 5-fluorouracil and doxorubicin have been shown to damage ovary and cause a reduction in bone volume[2,6,7]. Animal experiments with rats demonstrated that chemotherapeutic drugs increased bone resorption, while decreased bone formation[8]. However, there is no data whether chemotherapeutic drugs are effecting bone metabolism in early phase breast cancer interventions.

Breast cancer patients undergoing chemotherapy are routinely evaluated for their bone status by monitoring BMD and serum BTMs. BMD is measured every 6 month or 1 year intervals by DXA[9]. BTMs, which include markers for bone formation and bone resorption reflect the change in bone turnover state of individuals in a short time period[10–12]. These markers were used to predict the change in BMD and the risk of fracture, independently of BMD[13–15]. The reliability of previously identified BTMs such as serum ALP and urinary calcium is low. The recently identified BTMs such as PINP and BAP are more sensitive and reliable markers for bone formation. PINP specifically participates in type 1 procollagen synthesis and BAP is a tetrameric protein located on the plasma membrane of osteoblasts[16]. The β-isomer of β-Crosslaps and ICTP are sensitive and specific markers for pathological bone resorption. Another bone resorption marker, tartrate-resistant acid phosphatase isoform 5b (TRACP 5b) lacks specificity and sensitivity as a marker because of the presence of isoforms and the variation of their physiological expression[17]. The additional important factor for maintenance of skeletal health is vitamin D controlling bone mineralization[18]. Many studies observed a correlation between higher serum 25(OH)D and a reduction in hip-fracture risk[19,20]. PTH controls vitamin D1 level, as well as calcium and phosphate levels in the blood, and is important for regulating bone formation. An inverse correlation between 25(OH)D and PTH was observed among healthy Asian women[21]. Other important serum markers for the bone metabolism are E2 and FSH. E2 is essential for the maintenance of healthy bone structure and FSH is suggested to play a role in the regulation of bone density. It was shown that a chemotherapy-induced ovary failure led to decreased E2 and increased FSH serum levels in premenopausal women with breast cancer[22].

The previous study [23] focused on the long-term changes spanning 6 to 12 months in the serum BTMs of breast cancer patients undergoing chemotherapy. However, these changes might be caused by some other miscellaneous factors such as the tumor itself, the menopausal status of patients and other clinical interventions. Therefore, to evaluate the direct effects of neoadjuvant chemotherapy, real-time monitoring of the BTMs is required. In this present study, we performed a 1-year prospective study to assess the negative effects of chemotherapeutic reagents on the patients’ bone health and consequently promote the development of early clinical intervention for breast cancer patients.

## Patients and Methods

### Patients

Our study was a longitudinal observational study comprising 41 eligible preoperative breast cancer patients including 29 premenopausal and 12 postmenopausal women participated and were followed up for one year. The patients’ ages were between 30 to 64 years and they had a histologic diagnosis of invasive stageI,IIor III breast cancer. Women with a history of bone metabolic disease, hyperthyroidism, hyperparathyroidism, Paget’s disease, hypercalcemia, diabetes mellitus, hypertension, renal failure or distant metastasis were excluded from the study. Other clinical interventions with supplements such as bisphosphonate, calcium tablet and vitamin D at the time of study was also an exclusion criteria. As the control, age-matching 60 healthy women including 30 premenopausal and 30 postmenopausal were recruited. The menopausal status was defined by amenorrhea more than 3 months with FSH > 40 IU/L. This study was approved by the Institutional Ethic Committee of Peking University Cancer Hospital. All patients and healthy women signed the informed consent.

### Sample collection and measurement of serum BTMs concentrations

70.7% (29/41) patients received neoadjuvant chemotherapy with CEF/EC including cyclophosphamide 500mg/m^2^, epirubicin 100mg/m^2^, 5-fluorouracil 500 mg/m^2^, every 3 weeks, while the remaining 29.3% patients (12/41) were treated with EC-T (taxane 500mg/m^2^). Drugs were administered through intravenous infusion at the first and the eighth day in every chemotherapy treatment cycle. Blood samples were collected at 5 time points: the day of initial diagnosis before chemotherapy (C_0_), 24 hours after the first cycle of chemotherapy (C_1_), 24 hours after the second cycle of chemotherapy (C_2_), 24 hours after the third cycle of chemotherapy (C_3_), and 24 hours after the fourth cycle of chemotherapy (C_4_). The samples were processed immediately. 2 ml serum was prepared by centrifugation (1500g) for 10 minutes at room temperature and stored at -80°C until analysis.

Serum BAP and 25-(OH)D were measured using enzyme-linked immunosorbent assay (ELISA) (Immunodiagnostic Systems, Boldon, UK), and ICTP was measured by radioimmunoassay (RIA) (Orion Diagnostica, Espoo, Finland). The data were analyzed using a Multiskan Ascent analyzer (Thermo Fisher Scientific). Serum PINP, β-Crosslaps and PTH were quantified by electrochemiluminescent immunoassay using an Elecsys 411 automated analyzer (Roche Diagnostics, GmbH, Germany). Serum E2 and FSH were determined by electrochemiluminescent immunoassay using an Elecsys 170 automated analyzer (Roche Diagnostics).

The intra- and inter- assay coefficients of variation were less than 5% for the detection method of BAP and 25-(OH)D. The intra- and inter- assay coefficients of variation of the method for ICTP were less than 5.5% and 6.5%, respectively. The intra- and inter-assay coefficients of variation of the method for PINP, β-Crosslaps, PTH, E2 and FSH were less than 4% and 6%, respectively.

### BMD determinations

The BMD values of the lumbar spine (L1–L4) and one side of the proximal femur neck were determined with Prodigy DXA (GE/Lunar Co., USA) as T-scores. According to the WHO criteria, T-scores ≤ -2.5 at any skeletal site was defined as primary osteoporosis and T-scores between -1 and -2.5 as osteopenia.

### Statistical analyses

Statistical analysis was carried out using SPSS statistics for windows (version 17.0, Chicago, SPSS Inc). Differences in the levels of serum BTMs between the baseline and the respective time points (1st Cycle, 2nd Cycle, 3rd Cycle and 4th Cycle) were analyzed using the Wilcoxon matched-pair signed-rank test, and p<0.05 was considered to be statistically significant. Differences between the patient and the control groups were statistically analyzed using the Mann-Whitney U test. The correlations between any two markers were analyzed with the Spearman’s correlation test.

## Results

### Patients’ characteristics

From March 2013 to April 2014, 62 patients were registered in the study. Among them, five women were excluded from the study because of bone metastasis diseases, and 15 did not complete the 4 cycles of chemotherapy, therefore excluded from the analysis. The remaining 41 women (29 premenopausal and 12 postmenopausal) were assessable. For control, 60 healthy women (30 premenopausal and 30 postmenopausal) were enrolled in this study. The patient group and the healthy control group were matched with the age and the BMI. The characteristics of patients were shown in Table 1.

**Table 1:** Baseline characteristics of the patients (n = 41).

	Mean value ± SD
Average age (year) of	
Premenopausal patients	43±5
Postmenopausal patients	56±3
BMI (kg/m^2^)	24.2±3
	Number of patient (%)
Menopausal status	
Pre	29 (70.7)
Post	12 (29.3)
Stage of Breast cancer	
I	6 (14.6)
II	26 (63.4)
III	9 (22)
ER status ^a^	
Positive	25 (61)
Negative	16 (39)
HER-2 status ^b^	
Positive	33 (80.5)
Negative	8 (19.5)
Systemic adjuvant therapy	
CEF/EC	29 (70.7)
EC-T	12 (29.3)

C: cyclophosphamide, E: epirubicin, F: 5-fluorouracil, T: taxane.

^a^ER status: positive defined as McCarty's H score ≥50.

^b^HER2 status: positive defined by IHC≥3 or FISH amplified

### The effect of chemotherapy on the levels of the serum bone turnover markers in premenopausal women with breast cancer

**Fig 1:**
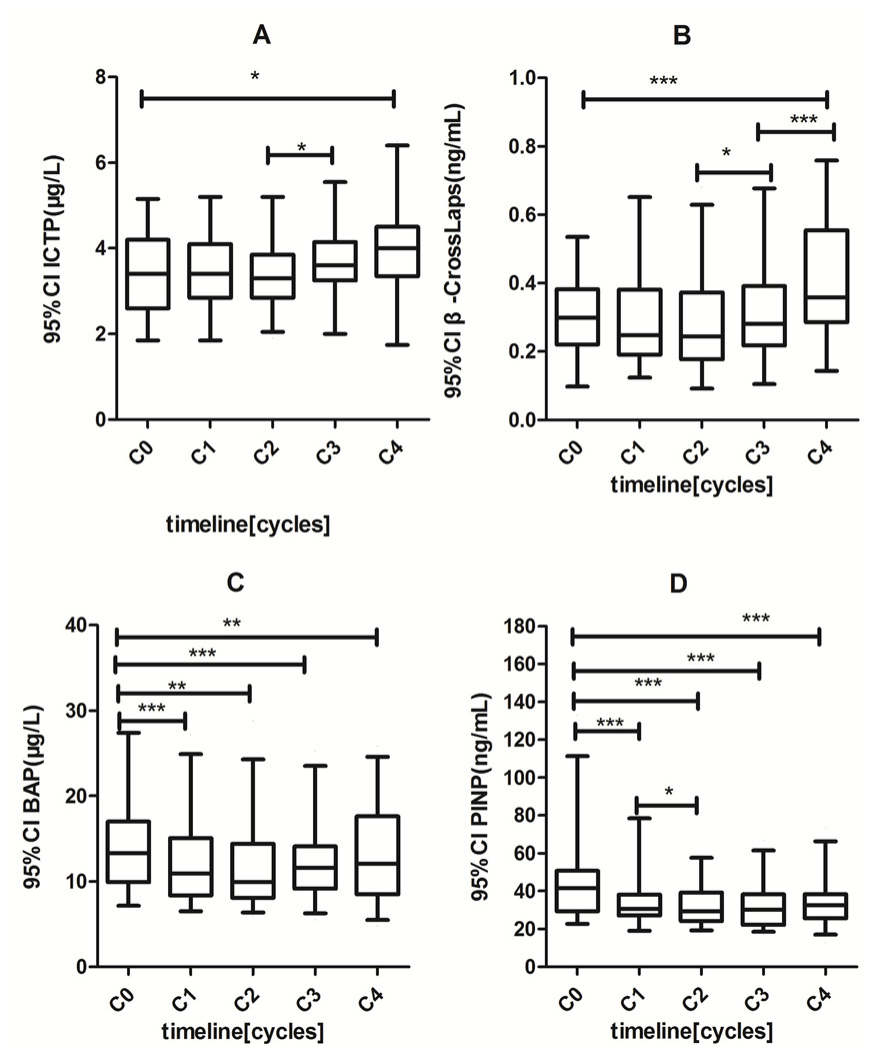
Changes of serum bone turnover markers levels under different cycles of chemotherapy in pre-menopausal breast cancers. (*) p<0.05; (**) p<0.01; (***)p < 0.001. C0: the day of initial diagnosis; C1–C4: 24 hours after the first, second, third. Fourth cycle of chemotherapy.

The serum levels of bone resorption markers ICTP and β-Crosslaps, and formation markers BAP and PINP in premenopausal patients before chemotherapy (C_0_), were significantly higher than those in premenopausal healthy women (p<0.05) (Table 2). During cycles of chemotherapy, the levels of ICTP and β-Crosslaps gradually increased, while the levels of BAP and PINP gradually decreased. The 95% confidence intervals (95% CI) of these changes were plotted in Fig 1. At the end of 4th cycle, the serum levels of ICTP and β-Crosslaps showed significant increase as compared to the baseline levels at C° from 3.39 μg/L to 4 μg/L (p<0.05) and from 0.300 ng/mL to 0.408 ng/mL (p<0.001), respectively. In contrast, the levels of serum BAP decreased from 14.5 μg/L to 13 μg/L (p<0.01) and PINP level decreased from 46.9 ng/mL to 34.01 ng/mL (p<0.001). Throughout 4 cycles of chemotherapy, the serum concentrations of these BTMs for both bone resorption and formation stayed considerably higher in premenopausal patients than in healthy premenopausal controls (p<0.01).

**Table 2:** The serum levels of BTMs in healthy women and in breast cancer patients prior to and after neoadjuvant chemotherapy.

	Control	C_0_	C_4_	P^a^	P^b^
**Premenopausal**					
ICTP (μg/L)	2.68±0.7	3.39±0.92	4±1.2	0.002^†^	0.028*
β-Crosslaps (ng/mL)	0.272±0.18	0.300±0.12	0.408±0.17	0.045*	0.000^†^
BAP (μg/L)	9.23±2.6	14.5±5.8	13±5.42	0.000^†^	0.001^†^
PINP (ng/mL)	27.3±7.7	46.9±24	34.01±12	0.000^†^	0.000^†^
**Postmenopausal**					
ICTP (μg/L)	3.36±0.6	2.95±0.7	3.8±0.9	0.105	0.034*
β-Crosslaps (ng/mL)	0.482±0.1	0.410±0.2	0.540±0.3	0.092	0.010*
BAP (μg/L)	16.6±3.0	17±4.1	13.94±4.7	0.824	0.028*
PINP (ng/mL)	61.06±19	63±29	47.75±20	0.867	0.012*

Data are reported as mean ± SD.

*Statistically significant at P < 0.05;

^†^Statistically significant at P < 0.01.

ICTP, carboxyterminal cross-linked telopeptide of type I collagen; β-Crosslaps, β-isomer of C-terminal telopeptide of type I collagen; BAP, bone-specific alkaline phosphatase; PINP, procollagen type I N-terminal propeptide.

C_0_, the initial diagnosis day before chemotherapy;

C_4_, 24 hours after the fourth cycle of chemotherapy.

P^a^: comparison between the healthy control and the patients at the baseline (C_0_).

P^b^: comparison between the baseline (C_0_) and after the 4th cycle of chemotherapy (C_4_) in patients

### The effect of chemotherapy on the levels of the serum bone turnover markers in postmenopausal women with breast cancer

**Fig 2:**
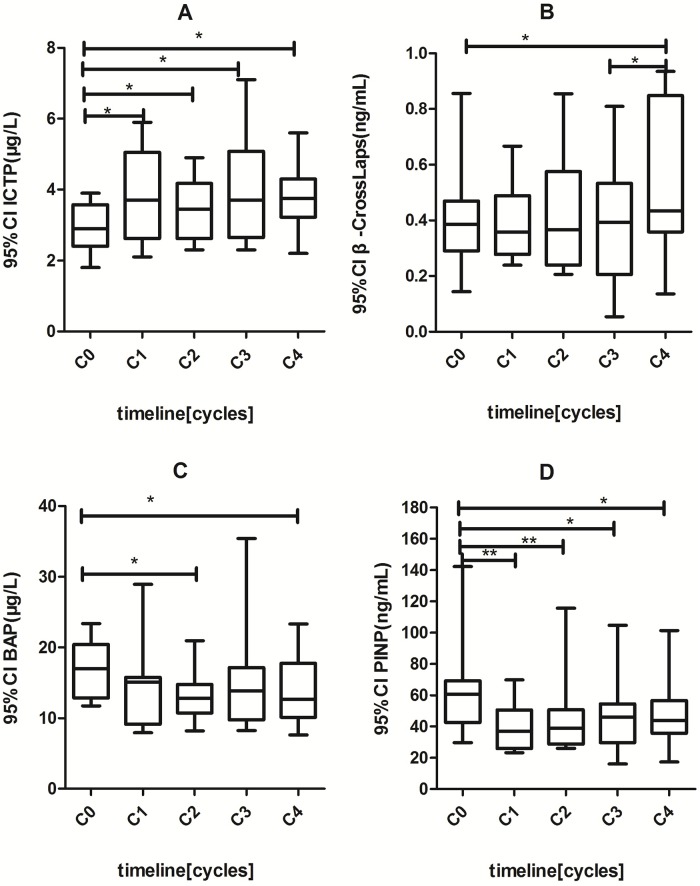
Changes of serum bone turnover markers levels under different cycles of chemotherapy in post-menopausal breast cancers. (*) p< 0.05; (**) p<0.01. C0: the day of initial diagnosis; C1–C4: 24 hours after the first, second, third. Fourth cycle of chemotherapy.

The serum BTMs In postmenopausal patients either at the C_0_ baseline levels or at the C_4_ post-chemotherapy levels showed no significant difference as compared to those in healthy postmenopausal women (Table 2). A comparison between the baseline levels and the levels during 4 cycles of neoadjuvant chemotherapy demonstrated that bone resorption markers, ICTP and β-Crosslaps significantly increased from 2.95 μg/L to 3.8 μg/L (p<0.05) and from 0.410 ng/mL to 0.540 ng/mL (p<0.05), respectively, while bone formation markers, BAP and PINP decreased from 17 μg g/L to 13.94 μg g/L (p<0.05) and from 63 ng/mL to 47.75 ng/mL (p<0.05), respectively (Fig 2). This pattern of increase and decrease in postmenopausal patients were similar to what was observed in premenopausal patients.

### Evaluation of the diagnostic sensitivity of serum BTMs combination measurements for abnormal bone metabolism

For a reliable diagnostic prediction of bone impairments, we analyzed the correlation among bone turnover markers. The biological variations within or between BTMs were reported to be less than 40%[24]. We set the threshold of the change rate as 50% for each metabolic indicator to determine the effect of chemotherapy. Among 41 patients who completed 4 cycles of chemotherapy, the levels of ICTP and β -Crosslaps were found increased in 12 (29.3%) and 13 (31.7%) patients, respectively. Combining the measurements of these two bone resorption markers, an increase was observed in 19 (46.3%) patients. The levels of bone formation markers BAP and PINP decreased in 6 (14.6%) and 13 (31.7%) patients, respectively. A combined decrease in these two markers was found in 18 (43.9%) patients. Consequently, combined measurements of bone formation and resorption markers demonstrated that chemotherapy altered the levels of BTMs in 25 (61%) patients.

### Effects of chemotherapy on the regulation of bone metabolism

**Fig 3:**
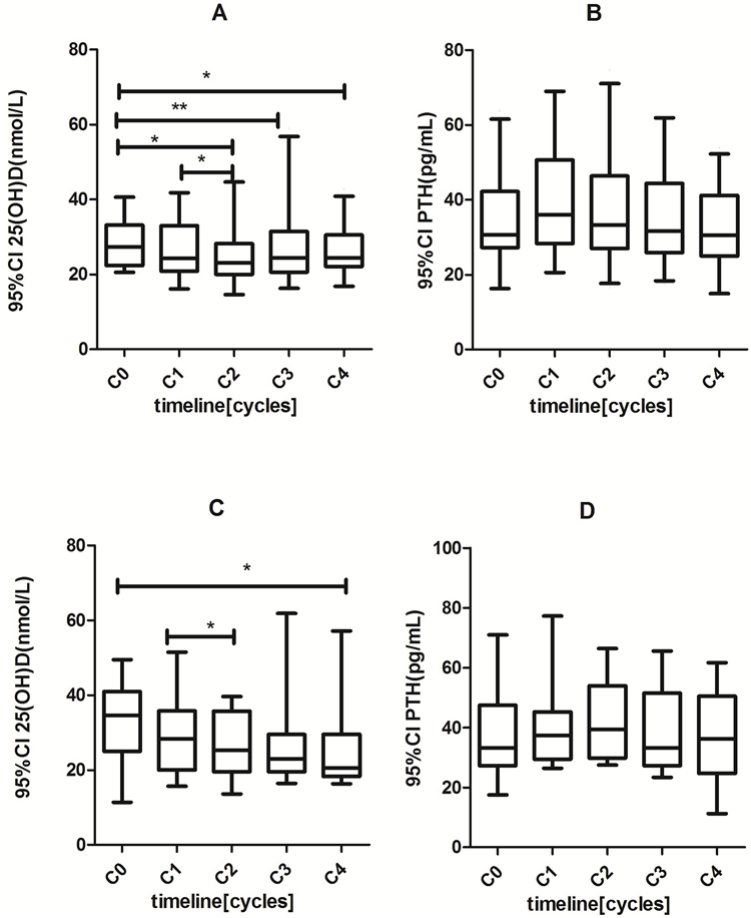
Changes of serum 25(OH)D and PTH levels under different cycles of chemotherapy in pre-menopausal (A and B) and post-menopausal breast cancers (C and D). (*) p<0.05; (**) p<0.01.

The levels of serum markers for the bone metabolism regulation in healthy women and breast cancer patients before and after 4 cycles of chemotherapy were demonstrated in Table 3. The level changes in these markers at the different time points of the chemotherapy cycles in pre- and postmenopausal cancer patients were depicted with 95% CI in Fig 3.

**Table 3:** Serum level changes of BTMs during the chemotherapy process.

	Control	C_0_	C_1_	C_2_	C_3_	C_4_	P^a^	P^b^
**Premenopausal**								
E2 (pg/mL)	159.8±117	148.6±160	60.2±56	31.5±30	11.3±10	7.1±3.1	0.203	0.00*
FSH (IU/L)	5.65±3.7	12±2.1	33.6±42	42.5±41	66.1±37	79.8±32	0.856	0.00*
25(OH)D (nmol/L)	38.6±7	28.3±6	25.6±6	12.4±5	11.8±5	12.0±4.3	0.000†	0.00*
PTH (pg/mL)	40.3±14.8	34.8±11	33±11	36±15	32.64±11	31.6±11	0.172	0.381
**Postmenopausal**								
E2 (pg/mL)	6.8±4.9	10.9±5	-	-	-	5.2±0.5	0.032*	0.01*
FSH (IU/L)	78.1±20	74.5±35	-	-	-	83.2±30	0.326	0.03*
25(OH)D (nmol/L)	39.1±5.2	32.8±11	25.6±12	29.3±10	26.8±8.6	27.1±13	0.051	0.02*
PTH (pg/mL)	42.8±14	37.4±15	36.5±15	40.3±15	41.4±13	38.8±15	0.248	0.346

Values were presented as mean± SD.

*Statistically significant at P < 0.05.

25(OH)D, 25-hydroxvitamin D; PTH, parathyroid hormone; E2, estradiol; FSH, follicle-stimulating hormone.

C_0_, the day of initial diagnosis before chemotherapy;

C_1_, 24 hours after the first cycle of chemotherapy;

C_2_, 24 hours after the second cycle of chemotherapy;

C_3_, 24 hours after the third cycle of chemotherapy;

C_4_, 24 hours after the fourth cycle of chemotherapy.

P^a^: comparison between the healthy control and the baseline of patients;

P^b^: comparison between the baseline (C_0_) and after the 4th cycle of chemotherapy (C_4_).

The baseline levels of serum 25(OH)D in premenopausal patients was lower than that in healthy premenopausal controls (p<0.001), while it had no difference between postmenopausal patients and postmenopausal healthy women. The analysis showed that cycles of chemotherapy caused a significant reduction in the levels of 25(OH)D both in premenopausal (p<0.01) and postmenopausal patients (p<0.05) (Fig 3).

With regard to PTH, we found no statistic differences between breast cancer patients at the initial diagnosis and healthy women. Regardless of the menopausal state, chemotherapy did not affect the serum PTH level (p>0.05) (Fig 3).

The serum levels of E2 and FSH in premenopausal breast cancer patients before chemotherapy showed no statistic differences from the healthy controls. However, chemotherapy caused an extremely significant decrease in E2, conversely, caused an extremely significant increase in FSH as compared with baseline (p<0.001). As a result, 4 cycles of chemotherapy generated a large disparity between patients and controls, the E2 level being much lower and the FSH level being much higher in premenopausal patients than in healthy women (p<0.001). In postmenopausal breast cancer patients, the serum E2 level significantly decreased while the FSH level significantly increased after chemotherapy as compared with baseline (p<0.05), (p<0.05). However, the changes were not as drastic as was observed in premenopausal patients, since the baseline level of E2 was already lower and that of FSH was higher in postmenopausal women than in premenopausal women.

### Comparison of CEF/EC and EC-T neoadjuvant chemotherapy regimen effects on bone metabolism marker

EC-T medication significantly reduced bone formation marker BAP (p<0.01) and PINP (p<0.01) serum concentrations compared with CEF/EC application, but the bone resorption marker ICTP and β-Crosslaps were not significantly different between the two regimens (Table 4).

**Table 4:** Percentage of BTM changes from baseline in the two groups of chemotherapy regimens.

	CEF/EC(n = 29)	EC-T(n = 12)	P-value
	mean, median		
BAP	9.52%, 10.3%	13.4%, 12.7%	p<0.01
PINP	13.5%, 17.6%	34.8%, 36.7%	p<0.05
ICTP	33.6%, 17.9%	10.8%, 1.6%	p>0.05
β-Crosslaps	40.9%, 22.6%	38.6%, 30.6%	p>0.05
25(OH)D	9.8%, 13.8%	10.3%, 25.8%	p>0.05
PTH	2.1%, 5.1%	9.5%, 5.3%	p>0.05

### Comparison of serum bone metabolism markers during neoadjuvant chemotherapy and BMD monitored 6 month later

BMD was determined 6 months after neoadjuvant chemotherapies in 12 of the 41 breast cancer patients, who received surgery. As shown in Table 5 the BMD T-scores of Patient 4,7,10 and 12 were less than -2.5, which indicated bone rarefaction. In these patients there was a tendency of particularly enhanced increases of bone resorption markers ICTP and β-Crosslaps combined with decreases of bone formation marker BAP and BMD after neoadjuvant chemotherapy 6 months before their BMDs were analyzed.

**Table 5:** Percentage of BTM changes after neoadjuvant chemotherapies in 12 patients and their BMD T-scores 6 months later.

Patient code	Baseline E2(pg/ml)	E2 after 6 months	ICTP increase	β-Crosslaps increase	BAP decrease	PINP decrease	BMD T-score
1 (pre)	134	15.00	113%	2%	-12%	68%	-2.0
2 (pre)	159	25.86	-16%	22%	15%	5%	-1.2
3 (pre)	219	7.40	67%	68%	6%	3%	-2.5
4 (pre)	53.03	6.70	117%	106%	11%	12%	-2.9
5 (pre)	186.2	34.2	-21%	106%	3%	-8%	-1.6
6 (post)	5.00	5.00	-35%	2%	-5%	43%	-2.0
7 (post)	5.50	5.00	97%	95%	17%	37%	-3.0
8 (post)	5.00	5.00	4%	57%	41%	59%	-2.0
9 (post)	5.74	5.00	27%	11%	13%	45%	-1.7
10(post)	5.60	5.00	139%	115%	34%	3%	-3.1
11(post)	5.00	5.00	18%	16%	-1%	18%	-1.8
12(post)	5.00	5.00	55%	51.5%	1%	29%	-2.6

pre: pre-menopausal, post: post-menopausal

## Discussion

In this study, the levels of BTMs in healthy postmenopausal women are evidently higher than those in premenopausal women, indicating that bone of postmenopausal women is in a high-turnover state. The levels of BTMs are negatively correlated with the level of estradiol, suggesting that down-regulation of estrogen is responsible for the high-turnover state of bone in postmenopausal women.

Our study showed that the levels of bone turnover markers in premenopausal women with breast cancer were significantly higher than those in healthy premenopausal women. Their levels were even similar to the levels observed in healthy postmenopausal women, demonstrating that the tumor itself might accelerate bone turnover, as reported by Ramaswamy *et al*. [3]. Probably this accelerated-bone metabolism was due to the increased levels of cytokines caused by tumor cells[25,26]. Tumor cells are able to produce factors that stimulate osteoclastic bone resorption directly or indirectly. These factors include IL-1, IL-6, PTHrP, prostaglandin E2, TNF, and CSF-1.[27]. Breast cancer cells can also produce IL-1, TNF and prostaglandins which increase RANKL expression and stimulate osteoclasts [28].

The levels of bone turnover markers in postmenopausal women with breast cancer were not apparently increased as compared with the healthy postmenopausal control group. We think that the main cause of bone loss observed in postmenopausal women with breast cancer is the decreased level of estrogen. This explanation is supported by the findings that the lower levels of serum E2 in postmenopausal women were associated with the higher rate of bone loss and the greater risk of fractures[28].

Our study demonstrated that neoadjuvant chemotherapy affected the bone metabolism in both premenopausal and postmenopausal women with breast cancer. The levels of bone resorption markers were found elevated in the first cycle of chemotherapy and further increased as the cycle of chemotherapy progressed. In contrast, the levels of bone formation markers significantly decreased after the first cycle, and then slightly increased after the third cycle. The result suggests that in women with breast cancer, chemotherapy induces menopause-like states with reduced serum concentrations of bone formation and elevated serum concentrations of bone resorption markers. Moreover, further analysis of specific neoadjuvant chemotherapy related effects on bone formation revealed, that Taxane has a stronger influence on BAP and PINP bone formation marker reductions than a CEF/EC regimen. Though BMD changes during neoadjuvant chemotherapies are not obvious, it is of interest, whether bone formation marker changes in the early medication period might indicate the risk of osteoporosis in later treatment periods. A postoperative BMD analysis 6 month after the neoadjuvant chemotherapies revealed that in deed patients with enhanced increases of bone resorption markers ICTP and β-Crosslaps combined with decreases of bone formation marker BAP and BMD had the tendency to develop osteoporosis. Among the 12 patients, the first 5 patients were premenopausal women and ICTP and β-Crosslaps of patient 3 and patient 4 were obviously increased by more than 50%, whereas BAP and PINP decreased somewhat. After half a year, BMD results indicated that 2 patients developed osteoporosis (T scores ≤ 2.5) and all others osteopenia (T scores between -1 and -2.5), which indicated that neoadjuvant chemotherapies can change bone turnover in premenopausal women and might be predictive for osteoporosis in a later phase. The latter 7 patients were postmenopausal women, in which ICTP and β-Crosslaps of patient 7, 10 and 12 also obviously increased more than 50% and BAP and PINP decreased to lesser extent. In this group 3 patients developed osteoporosis and all others osteopenia, indicating that in post-menopausal women the bone turnover changes after neoadjuvant chemotherapies were similar to pre-menopausal women but more pronounced 6 months after medication (Table 5).

The effects of neoadjuvant chemotherapies might further be explained by the following possibilities: 1. Chemotherapy directly promoted the osteoclast activity; 2. Chemotherapy inhibited estrogen secretion from ovary resulting in an indirect increase in the osteoclast activity. 3. Cytokines, which were released by apoptosis of tumor cells induced by chemotherapy, stimulated the osteoclast activity. 4. Chemotherapy directly inhibited the activity of osteoblasts. In addition, the United States Endocrine Society guidelines recognize the serum concentration of 25(OH)D less than 30ng/mL as a vitamin D deficiency[29]. In our study, four (6.67%) women in the healthy group, while 28 (68.3%) in the patient group were deficient in vitamin D. Some epidemiologic and observational data suggested cancer could cause a reduction in the serum 25(OH)D level[30]. In our study, the serum 25(OH)D level in premenopausal patients showed a constant and significant decrease during cycles of chemotherapy with the reduction range of 58.5%. There was no significant correlation between either 25(OH)D or PTH and the bone turnover markers PINP and β-Crosslaps, corroborating the result obtained by Tan et al.[21]. However, Vitamin D promotes absorption of calcium and phosphate and thereby bone formation. Therefore, we suggest that the decreased level of vitamin D by the chemotherapy cycles might also be a factor for an increase in bone resorption markers and conversely a decrease in bone formation markers observed in breast cancer patients.

A combined measurement of several bone turnover markers give rise to a more accurate estimate of metabolic bone impairments than a single marker measurement, because in our study, the multi-marker combination measurements identified chemotherapy effects in 61% of the patients compared to 14.6% with single marker measurements.

Limitations of our study included the lack of breast cancer related cytokine expression measurements and analysis of their possible influence on bone formation marker. In addition for more accurate predictions of osteoporosis risk, a study involving a larger number of patients and a longer-term follow-up has to be carried out and the simultaneous measurement of bone turnover markers and bone mineral density has to be extended to cover the entire process of neoadjuvant chemotherapy and treatment after surgery.

In summary, our results revealed that neoadjuvant chemotherapies of breast cancer patients led to reductions of bone formation marker and enhancements of bone resorption marker serum concentrations, the latter one more pronounced with Taxane medication. Particularly patients with pronounced ICTP and β-Crosslaps combined with reduced BAP and PINP serum concentration increases during neoadjuvant chemotherapy were prone to develop osteoporosis 6 month later. Since 25(OH)D serum concentrations declined significantly during neoadjuvant chemotherapies in premenopausal and postmenopausal breast cancer patients, we suggest to provide a timely care for breast cancer patients undergoing chemotherapy with an adequate dose of vitamin D as well as instructions for sunbath and weight-bearing exercises.
